# Effect Mechanism of Error Management Climate on Innovation Behavior: An Investigation From Chinese Entrepreneurs

**DOI:** 10.3389/fpsyg.2021.733741

**Published:** 2021-12-07

**Authors:** Yuting Chen, Jiangru Wei, Jing Zhang, Xue Li

**Affiliations:** ^1^School of Management, Nanjing University, Nanjing, China; ^2^School of Management, Nanjing University of Posts and Telecommunications, Nanjing, China

**Keywords:** error management climate, entrepreneurial self-efficacy (ESE), innovation behavior, Zhongyong thinking, error learning

## Abstract

Errors are inevitable in an increasingly risky and dynamic entrepreneurial environment. The error management and the error climate perceived by the members are crucial to the subsequent innovation behaviors. Maintaining and improving the psychological capital of entrepreneurs under errors is not only the psychological activities of entrepreneurs themselves but also a critical management process in which an organization can influence the psychological factors and behaviors of entrepreneurs through error management climate. In the context of Chinese culture, this study explores the influence of error management climate on entrepreneurial self-efficacy and innovation behavior under the boundary condition of Zhongyong thinking. Two hundred ninety samples of Chinese entrepreneurs are empirically analyzed in this study, and results show that: (1) error management climate and entrepreneurial self-efficacy have significant positive effects on entrepreneurs’ innovation behavior; (2) entrepreneurial self-efficacy mediates the relationship between error management climate and innovation behavior; and (3) Zhongyong thinking plays moderating roles in the process of error management climate influencing innovation behavior. This study complements the entrepreneurship literature with its focus on error management climate as an essential antecedent of entrepreneurial self-efficacy, and promotes an understanding of how Chinese practitioners promote innovative behavior from a cultural perspective.

## Introduction

With the increasing dynamics and uncertainty of the entrepreneurial environment, entrepreneurs face more difficulties in the entrepreneurship, and errors are never utterly avoidable because of the limitations and imperfections in the practice (Khelil, 2016). Causes of errors may include fatigue, fear, limited cognition, incomplete information, and flawed decision-making (Fruhen and Keith, 2014). Researchers advocate a systematic review of success and focus more on the information conveyed by the error experience (Yamakawa and Cardon, 2014). Errors in the entrepreneurship process bring a range of negative consequences, but can also be excellent opportunities for organizational learning and innovation (Headd, 2003). Literature on innovations implies that errors can be part of developing innovation and bringing it to the market (Hsu et al., 2015). Increasing corporations take errors as part of climate management, and how they perceive errors will affect reaction to errors (Rupert et al., 2019).

According to how the organization deals with errors and their consequences after they occur, error climate is divided into positive and negative error climates (van Dyck et al., 2005). Action-oriented error management climate (EMC) belongs to positive error culture, and EMC aims to control the adverse effects of errors and promote their positive effects. Companies advocating EMC tolerates errors and emphasizes their learning function, making employees regard errors as part of their work and pay more attention on possible implementation paths or methods to deal with errors (Zhang and Song, 2020). On the contrary, the emotion-oriented error aversion climate is negative error climate. An organization with a climate of error aversion has a low tolerance for errors, leading entrepreneurs to try to avoid errors and tend to behave conservatively in the workplace (Fruhen and Keith, 2014). Once error occurs, employees will cover up the error to maintain their self-esteem and reduce organizational punishment (van Dyck, 2009).

The study of error management first emerged in the aviation and medical industries, which are sensitive to error at the end of the 20th century (Finkelstein et al., 1997), further developed into organizational behavior because of its significant role in predicting employee behavior and performance (Frese and Keith, 2015; Wang et al., 2020). However, there are few studies on EMC in entrepreneurship and how EMC affects entrepreneurs’ attitude and response to errors remains further understanding. The way and behavior of entrepreneurs to recognize, treat, and prevent errors affect their innovative activities, and the management of errors has been neglected in the existing entrepreneurial research. In order to explain how errors become the source of innovation, this paper attempts to explain the process of EMC influencing innovation behavior (IB) in the entrepreneurship field.

Conservation of resources theory (COR) holds that individuals tend to preserve, protect, and acquire resources that are valuable to them for accomplishing goals (Hobfoll, 2001). Hobfoll (1989) distinguished four types of resources, including individual-specific resources, such as high intelligence, optimism, and self-efficacy. As the application of self-efficacy in entrepreneurship research, entrepreneurial self-efficacy (ESE) is regarded as an important personal resource for entrepreneurs and plays a vital role in predicting behaviors associated with innovation (Barakat et al., 2014). Entrepreneurs with a great sense of ESE are more confident in achieving innovation goals and more likely to overcome difficulties in the process of innovation (Ahlin et al., 2013). Risky innovation requires entrepreneurs to have adequate psychological capital to reduce stress (King and Beehr, 2017), and ESE can be considered an important intangible resource related to the realization of entrepreneurial activities. Thus, the innovation behavior of entrepreneurs can be promoted, firstly, through the improvement of ESE. In order to protect and acquire ESE, conservation of resources theory also advocates to create and maintain a positive and healthy work climate (Hobfoll, 2001), implying that EMC may be a predictor of promoting ESE. EMC encourages entrepreneurs to consider the error situation as an opportunity to improve the error capacity through error thinking, error communication, and error learning, which helps consolidate and improve ESE (Raub and Liao, 2012).

Theoretical thinking on whether and how national culture factors affect entrepreneurial activities have been discussed for almost a century. Interestingly, a large number of countries with collectivist cultures have witnessed much more active entrepreneurial activities than many Western countries with individualistic cultures in recent 20 years, arousing scholars’ attention on the cultural factors behind entrepreneurial activities in developing countries. Since this study is based on the Chinese context, the personal characteristics under the broader cultural environment of Chinese entrepreneurs need to be further considered. Zhongyong thinking (ZYT) is a representative cultural capital and traditional value concept in Chinese Confucianism, which profoundly impacts the thinking mode and behavior style of the Chinese people (Zhou et al., 2019). Series of studies advocate that ZYT contributes to a more optimistic attitude, harmonious interpersonal relationships, and flexible ways of doing things in terms of self, interpersonal, and work (Qu et al., 2018). Recent evidence also suggests that ZYT can help people adjust their beliefs and behaviors to develop more effective coping strategies in a dynamic and complex entrepreneurial environment (Wei et al., 2020). ZYT is a typical cognitive attitude adopted in social interaction in Oriental culture, and regarded as an important boundary condition in this study.

Therefore, the purpose of this study is to answer and provide empirical evidence on how EMC influence entrepreneurs’ IB in the Chinese context. Based on the conservation of resources theory (COR), this study investigates whether the EMC is an important factor in translating errors into the psychological capital of entrepreneurs and effectively carrying out innovative behaviors from the perspectives of ESE and ZYT. This paper answers how does EMC influence innovation behavior by explaining the beneficial effect of EMC, indicating that EMC is beneficial to reasonable errors handling and conducive to innovation behavior. Then, ESE is introduced as an essential psychological resource, expanding the application of the theory in the management of entrepreneurial errors. Finally, this paper provides empirical experience from China on how ZYT plays a positive moderating role in both direct and indirect relationships between EMC and IB, contributing to the understanding of the thinking characteristics of Chinese entrepreneurs.

## Theory and Hypotheses

### Error Management Climate and Innovation Behavior

van Dyck et al. (2005) proposed EMC as a climate in which employees have the common perception of the practice and behavior of the organization. Taking measures to reduce the negative consequences of errors and increase the potential positive consequences is an important practical content (Fischer et al., 2018). Based on the positive error perception perspective, organizations with a high EMC believe that errors are valuable (Shepherd et al., 2009). There are a series of relevant practices in the organization for error analysis, error communication, error learning, and error ability, promoting the understanding of errors among entrepreneurs, and effectively avoid more significant losses in the future (Byrne and Shepherd, 2013).

Recent studies have found that EMC positively impacts subsequent entrepreneurial performance (Hmieleski and Corbett, 2008). In order to gain competitive advantage, innovation ability and behavior of entrepreneurs are critical for development potential of new enterprises. From the perspective of process view, innovation is about analyzing and solving problems (Sternberg, 2006). IB refers to the behavior that individuals generate new ideas or solutions after identifying and recognizing problems, and to further promote the application in the organization by seeking support and recognition (Scott and Bruce, 1994; Wu et al., 2011). Research on the antecedents of innovation behavior has been explained by entrepreneurs’ traits, knowledge and skills, and emotional state (Yao et al., 2010; Xerri, 2014; Tang and Ye, 2015), but there is still a lack of research on the perspective of EMC in the field of entrepreneurship.

Innovation process is uncertain and errors are hard to avoid altogether. Attitude to errors and how to deal with them in the enterprise affect entrepreneurs’ expectations of innovation results. When errors occur, the management climate encourages entrepreneurs to view errors as an important resource, thus improving their ability to cope with errors and reducing the loss subsequently (Shepherd et al., 2009). EMC is conducive to an atmosphere of critical thinking and open discussion of entrepreneurial problems, making entrepreneurs more willing to conduct error analysis and expose their personal mistakes in a team environment. When entrepreneurs are not bothered by the negative consequences of errors, they can allocate more resources to solving constructive problems in the innovation process (Bradley et al., 2012). Secondly, a higher EMC promotes error communication. It enables entrepreneurs to freely share consensus and knowledge about errors, which deepens the understanding of the innovation process and enhances entrepreneurial teams’ mutual assistance and collaboration ability (Keith and Frese, 2005). Thirdly, enterprises with a high EMC encourage error recognition and error learning, contributing to the ability to handle errors (Tobin et al., 2006) and the adaptability and confidence of entrepreneurs. Therefore, EMC helps transform errors into valuable resources for entrepreneurs, and the accumulation of error resources is conducive to innovation activities. Thus, this paper reaches the following hypothesis:

H1: EMC has a positive effect on entrepreneurs’ IB.

### Error Management Climate and Entrepreneurial Self-Efficacy

Entrepreneurial self-efficacy (ESE) is a concept applied in entrepreneurship from Bandura’s research. ESE refers to the self-confidence intensity of entrepreneurs on whether their entrepreneurial skills can complete various entrepreneurial activities and behaviors (Boyd and Vozikis, 1994; Chen et al., 1998). ESE also emphasizes the subjective initiative of the individual and plays a vital role in self-recovery and self-motivation, indicating the degree of confidence of the individual rather than just the ability itself. The promotion and enhancement of ESE has become the psychological construction in overcoming various difficulties and achieving entrepreneurial success (To et al., 2020).

Based on conservation of resources theory (COR), individuals tend to acquire four kinds of resources that are valuable to them, one of which is individual characteristic resources, such as intelligence and optimism (Hobfoll, 1989). ESE is an important psychological resource for entrepreneurs, which is beneficial to opportunity identification, risk-taking and innovation activities related to entrepreneurship (Li et al., 2020). In order to protect and acquire ESE, COR also provides guidance for individuals in stressful situations and prevention in advance, that is, to create and maintain a positive and healthy work climate (Hobfoll, 2001). EMC is a positive climate that advocates error analysis, error communication, and learning, implying that EMC may play a positive role in the acquisition and protection of ESE. Through mutual information sharing, they cannot only improve the identification, prevention and timely correction of errors, but also promote dynamic behavior of error learning to enhance the courage and confidence of self-exploration and innovation continuously (Ibrayeva, 2006). EMC encourages entrepreneurs to take error situations as an improvement opportunity, contributing to risk-taking in entrepreneurial activities and possibly find the path to success (Kasouf et al., 2015). Therefore, ESE can be consolidated and enhanced under EMC.

H2: EMC has a positive effect on the ESE of entrepreneurs.

### The Mediation Effect of Entrepreneurial Self-Efficacy

Previous studies have explained how to improve innovation behavior from entrepreneurial climate and psychological capital (Downes et al., 2016). However, there is a lack of discussion on how EMC influences innovation behavior and research evidence from Chinese entrepreneurs. Taking the resource conservation theory as the cognitive lens, unique resources can be nurtured and nourished by situational characteristics and affect individual behaviors (Hobfoll, 2001). This paper holds that ESE is an important mediating variable that conducts perceived environmental factors to individual innovation behaviors.

As a valuable intangible resource for the individual, ESE has been explained its positive role in realizing a series of entrepreneurial goals (Chen and Zhou, 2017). Innovation is a desirable but difficult goal for entrepreneurs, and the process is full of risks and uncertainties. Existing research has found that people with higher ESE are more capable of dealing with this reality (Mcgee and Peterson, 2017). Entrepreneurs with high ESE have more positive expectations of results than those with low ESE to set appropriate innovation goals and practices, and the same entrepreneurial environment can be evaluated as full of opportunities (Caines et al., 2019). Thus, entrepreneurs with a high sense of ESE have strong confidence in forming innovative behaviors. They will fully invest in and constantly work hard to overcome difficulties in the innovation stage, which in return ESE also be modified and reinforced. However, individuals with low ESE are prone to avoid problems or even quit in facing entrepreneurial difficulties, mainly when emotional exhaustion is often caused in the process of entrepreneurship (Wei et al., 2019). As discussed above, EMC improves ESE through error thinking, error communication, and error learning. Furthermore, ESE helps entrepreneurs to overcome difficulties in innovation activities and achieve innovation goals. Therefore, EMC can positively influence innovation behavior by promoting ESE of entrepreneurs. This study proposes that:

H3: ESE mediates the relationship between EMC and IB.

### Moderating Effect of Zhongyong Thinking

Originating from Confucian philosophy, ZYT is a cognitive thinking about how Chinese think about objects, people, and the environment (Pierce and Aguinis, 2013). ZYT has a subtle influence on Chinese attitudes and behavior for thousands of years as they are likely to avoid an extreme perspective when confront with contradictions and conflicts, and more inclined to choose a moderate way (Lee, 2000). ZYT refers to a thinking mode about integrating both external conditions and internal needs from multi-perspectives and taking practical actions in a specific situation (Wu and Lin, 2005). Core principles of ZYT include (1) multi-thinking, which emphasizes looking at problems with dialectical thinking and understanding things from multiple angles, (2) integration, requiring the individual to take the external changing situation into the consideration of the internal thinking, and (3) harmoniousness, referring to the degree to which actions are conducted in a concordant method (Wu and Lin, 2005; Chang and Yang, 2014). Although similar concepts can be found in western theories, such as dialectical thinking, rationality, and wisdom (Peng and Nisbett, 1999), ZYT represents a set of unique life concepts and worldview of Chinese people, involving various aspects of self, interpersonal relationship, and dealing with affairs. People with ZYT aim to achieve harmonious goals and make choices out of the most suitable way after considering internal and external conditions (Yang et al., 2016). ZYT implies the idea of making progress with environment and time, which is not only one of the cultural characteristics of the Chinese people, but also a cognitive strategy to effectively cope with today’s changing and uncertain environment (Wei et al., 2020).

Based on COR, situational characteristics can play a nurturing role in fostering personal resources and, on the other hand, they may have a negative effect, implying that broader cultural environment of the entrepreneur plays an important role in shaping and maintaining resources (Hobfoll, 2001). As a thinking characteristic formed under the Confucian culture, ZYT affects the attitude and behavior of Chinese entrepreneurs in integrating resources and facing risks in entrepreneurship. Chou et al. (2014) demonstrated that ZYT can be an effective cognitive strategy for coping with work stress. Thus, ZYT can be considered as a cognitive strategy that displays positive effects of EMC on ESE. Firstly, entrepreneurs with a higher degree ZYT tend to be multi-thinking and consider error situations from a long-term perspective, which means they are less likely to be biased by the negative emotions for a moment. Multi-thinking helps to weaken the contradiction and adapt to environmental changes in the process of innovation activities. Secondly, the holism of ZYT encourages entrepreneurs to combine objects, people, and environment together, which contribute to integrating various resources (Wu and Lin, 2005). Entrepreneurs with a ZYT constantly think, learn, and optimize to communicate and learn from errors to carry out innovation behavior effectively. Thirdly, ZYT is conducive to achieve innovation goals by choosing the most suitable way after considering various factors. That is, entrepreneurs with higher-level ZYT can use self-consistent methods to conduct innovative behavior. Therefore, this study develops the following hypothesis:

H4: Relationship between EMC and IB is moderated by ZYT. Hence, the positive relationship between EMC and IB is stronger among entrepreneurs with higher ZYT than those with lower ZYT.

Zhongyong thinking (ZYT) is a typical thinking mode of Chinese, which affects entrepreneurs’ attitude, psychology, and behavior. Thus, this paper studies whether ZYT has a moderating effect on the relationship between ESE and IB. First of all, people with high ZYT recognize the objects and people in a multi-dimensional way, and they also identify their own personalities as contradictory factors in order to keep more flexible beliefs and behaviors to meet the changed context (Chen, 2005), which allows entrepreneurs to focus on innovation activities. Secondly, entrepreneurial activities are constantly changing, and individuals with high ZYT can change their cognition and behavior into a new environment. In this case, entrepreneurs have confidence in integrating various resources and striving to translate personal beliefs into innovative activities. Thirdly, the pursuit of interpersonal harmony is a kind of “harmony in diversity.” In innovation activities, entrepreneurs are good at expressing opinions and euphemistically persuading others, and are expected to seek entrepreneurial support to achieve innovation goals. Previous studies have shown that entrepreneurs are more willing to carry out innovative behaviors when individuals have a high belief in innovative activities (Duan et al., 2010; Zhou et al., 2020). Therefore, this study believes that entrepreneurs with Zhongyong thinking have higher flexibility, confidence, and positive expectation of results, representing a higher level of ESE, further promoting entrepreneurs’ innovation behaviors.

**FIGURE 1:**
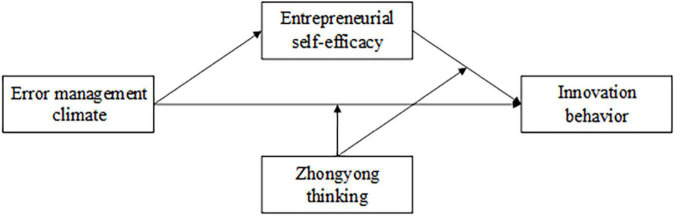
Research framework.

Zhongyong thinkers believe that the world is always changing and full of contradictions, and they are more focused on the circumstances and relationship of the object. High Zhongyong thinkers understand that error and entrepreneurial success are inseparable, and they realize sustainable entrepreneurship by constantly learning and reflecting from errors. Perceiving a good EMC, entrepreneurs with higher ZYT can quickly conduct error reflection and learning from various angles and actively reduce the damage of errors to entrepreneurial activities (Arts et al., 2011). At the same time, entrepreneurs can summarize more experience from the errors and have more confidence to meet the challenges in the future. It is conducive to the accumulation of ESE as psychological capital, which is further beneficial for entrepreneurs to adopt appropriate ways to carry out entrepreneurial activities. ZYT enhances the process of ESE influencing IB, and thus enhance the mediating role of ESE between EMC and IB. Thus, this study develops the hypotheses and proposes a conceptual model (Figure 1).

H5a: ZYT moderates the direct relationship between ESE and IB; the positive relationship between ESE and IB is stronger among entrepreneurs with higher ZYT than those with lower ZYT.
H5b: ZYT moderates the indirect effect of EMC on IB through ESE, such that the mediated relationship is strengthened when an entrepreneur has a higher level of ZYT.

## Materials and Methods

### Participants and Procedures

This research conducted questionnaire surveys to collect information from Chinese participants, and the hypotheses were tested by the multiple regression analysis methods. Given that supportive policies encourage business incubators to provide physical space and infrastructure for new technology-based small and medium-size enterprise in China, we chose a business incubator to conduct a questionnaire survey. Through an entrepreneurship center that corporations closely with the incubator, we firstly contacted an incubator manager and invited entrepreneurs to participate in the online questionnaire. The email stated the purpose of the survey and ensured that the questionnaire was anonymous and did not involve any commercial interests. It also emphasized that all participants could withdraw at any time during the filling process, so that interested people could participate in the survey by clicking on the link provided in the email.

The start-ups are mainly engaged in electronic information, internet, and financial service industries, which are representative samples for the research on innovation behavior (Wei et al., 2020). We distributed 400 questionnaires in this study, and 327 questionnaires were given feedback. Thirty-seven questionnaires were eliminated due to abandonment or omission, and we obtained 290 valid questionnaires. As illustrated in the Table 1, the sample consisted of 153 males (52.76%) and 137 females (47.24%). Respondents are mainly under the age of 40 (*n* = 259, 89.32%) and most of them have a bachelor’s degree (*n* = 179, 71.72%). Eighty-five (29.31%) participants worked less than 1 year in the startup, 72 (24.83%) had 1–3 years working experience, and 133 (45.86%) participants had worked for more than 3 years.

**TABLE 1:** Sample description (*N* = 290).

Individual characteristics	Category	Quantity	Percentage
Gender	Male	153	52.76%
	Female	137	47.24%
Age	≤25	84	28.97%
	26–30	99	34.14%
	31–35	51	17.59%
	36–40	25	8.62%
	≥41	31	10.68%
Education background	High school and below	10	3.54%
	Diploma	19	6.55%
	Bachelor	179	61.72%
	Master and above	82	28.29%
Experience in the start-up	≤1 year	85	29.31%
	1–5 years	72	24.83%
	≥5 years	133	45.86%

*The tail difference of percentages is adjusted at the end of each item.*

### Measurement of Variables

This study adopted five-point Likert scales to measure EMC, ESE, IB, and ZYT. Control variables were converted to dummy variables, and all items in the questionnaire were self-reported by the entrepreneurs.

#### Error Management Climate

We adopted the scale developed by van Dyck et al. (2005) to measure the EMC, and seven items were used. The items mainly include the entrepreneur’ positive perception of errors and their tendency to learn from errors. Representative items state as “After making a mistake, people try to analyze what caused it.”

#### Entrepreneurial Self-Efficacy

Following the studies of Mcgee et al. (2009) and Barakat et al. (2014), four-question items were adopted to measure ESE. Sample items include “I can choose suitable employees for my business,” “I can come up with new ideas to solve problems in entrepreneurship,” and “I have confidence in my ability to solve problems in my business.”

#### Innovation Behavior

Single-dimensional scale introduced by Scott and Bruce (1994) were widely accepted and used with its good reliability and validity (Kang et al., 2016), and we also used the six items to measure IB. The representative items of the scale are “I always seek to apply new processes, techniques and methods” and “In order to implement new ideas, I can find ways to get the resources I need.”

#### Zhongyong Thinking

Based on the definition and connotation of ZYT, this paper measures ZYT with reference to the research of Wu and Lin (2005). The multi-thinking dimension included four items, such as “When I make a decision, I will consider various possible conditions.” The integration dimension consists of five items, such as “I often try to find acceptable opinions in a situation of disagreement.” The harmoniousness dimension includes four items, such as “I usually adjust my behavior for overall harmony.”

#### Control Variables

Previous empirical studies have shown that gender, age, education background, and experience in the start-up are related to IB (Wei et al., 2020). In order to exclude possible alternative explanations, the above four demographic variables were set as control variables and dummy variable assignment method was adopted in this study.

### Common Method Bias Control

We strictly follow the principles of confidentiality and voluntary to control the bias in research design as procedural remedies. Meanwhile, questionnaires are collected immediately *via* the link in emails after completion to ensure that the data are not modified. In addition, this paper also uses the commonly accepted Harman single factor analysis method to test common method biases. It is acceptable that the number of factors extracted is more than one and the variance contribution rate of the first factor is not more than 40%. In that case, it is generally considered that the deviation of the common method is not severe. Harman single factor test showed that the four factors of principal component analysis explained 63.40% of the total variance, of which factor 1 explained 30.11%. Therefore, standard method bias has not significant affect on this study.

### Confirmatory Factor Analysis

Confirmatory factor analysis (CFA) was conducted to evaluate the discriminant validity of factor combinations in the fitting model and determine whether the four-factor model hypothesized in this study is the best combination (Table 2). We adopted five indicators commonly used in empirical studies to test the discriminant validity of the four-factor model, including the chi-square degree of freedom ratio (χ^2^/df), root mean square error of approximation (RMSEA), root mean square residual error (RMR), the comparative fit index (CFI), modified fit index (IFI), and Tuck–Lewis index (TLI) of the relative fitting index. According to the index evaluation criteria recommended by Browne and Cudeck (1992), when χ^2^/df is between 1 and 3, RMSEA is less than 0.08, RMR is less than 0.05, CFI, IFI, and TLI are greater than 0.9, the fitting effect is acceptable. Compared with the alternative models (M1 ∼ M5), the hypothesized four-factor model (M0) in this study displayed the best fit with the data (χ^2^/df = 1.769, CFI = 0.931, IFI = 0.920, RMSEA = 0.051).

**TABLE 2:** Confirmatory factor analysis by comparing alternative measurement models.

Model	Description	χ^2^	*df*	CFI	IFI	RMSEA	Δχ^2^
M0	Four-factor model (EMC, ESE, IB, and ZYT)	705.650	399	0.931	0.920	0.051	–
M1	Three-factor model (EMC, ESE + IB, and ZYT)	1052.819	402	0.854	0.832	0.074	347.169***
M2	Three-factor model (EMC, ESE, and IB + ZYT)	1158.648	402	0.831	0.804	0.080	452.998***
M3	Three-factor model (EMC, ESE + ZYT, and IB)	1187.447	402	0.824	0.797	0.081	481.797***
M4	Two-factor model (EMC and ESE + IB + ZYT)	1606.802	404	0.731	0.690	0.100	901.152***
M5	One-factor model (EMC + ESE + IB + ZYT)	2454.929	405	0.541	0.474	0.131	1749.279***

N = 290; ***p < 0.01, 2-tailed.

### Reliability and Validity of the Scales

Before testing the proposed conceptual model, Cronbach’s α was used to test the reliability of the questionnaire. When Cronbach’s α is higher than 0.7, the reliability of the scale is good and the scale can be accepted (Kline, 1998). Results showed Cronbach’s α of EMC, ESE, IB, and ZYT in this study are 0.821, 0.841, 0.849, and 0.914, indicating that scales used in this study are acceptable. According to the study of Fornell and Larcker (1981), the convergence validity of the scale can be judged by the standardized factor loading, average variance extraction (AVE), and combined reliability (CR) of each item. When the results meet the following three conditions, it indicates that the scale has good convergence validity. First, the standardized factor loading of each item is greater than 0.5 and significant. Second, AVE represents the interpretation rate of cumulative variance of construct items, and 0.5 is generally used as the threshold in studies. Third, the value of CR is greater than 0.7. Appendix Table 1 showed the standardized factor loading of each factor exceeds 0.6. AVEs were significant at a 0.001 significance level and all exceeded 0.5. The CR of EMC, ESE, IB, and ZYT are 0.956, 0.860, 0.939, and 0.967, respectively. Therefore, the reliability and validity of the scales are good, and the subsequent data analysis can be carried out.

### Descriptive Statistics and Correlations

In order to analyze whether the control variables have a significant impact on the main constructs and whether there is a significant correlation between the main constructs, Pearson correlation coefficient analysis was conducted and results are shown in Table 3. EMC has a significant positive relationship with IB (β = 0.548, *P* < 0.01), and also positively relates to ESE (β = 0.355, *P* < 0.01). These results provide preliminary support for subsequent hypothesis testing.

**TABLE 3:** Descriptive statistics and correlations among the variables.

Variables	Mean	SD	1	2	3	4	5	6	7	8
(1) Gender	1.47	0.50								
(2) Age	2.38	1.28	−0.130*							
(3) Education background	3.15	0.68	0.058	−0.342**						
(4) Experience in the startup	2.17	0.85	−0.176**	0.326**	−0.340**					
(5) Error management climate	3.54	0.73	0.001	0.034	–0.097	0.030	**0.757**			
(6) Entrepreneurial self-efficacy	3.74	0.57	–0.065	0.072	–0.003	0.021	0.355**	**0.735**		
(7) Innovation behavior	3.77	0.46	–0.016	0.009	–0.027	−0.038	0.548**	0.359**	**0.674**	
(8) Zhongyong thinking	3.96	0.42	–0.033	0.000	–0.006	0.036	0.301**	0.190*	0.503**	**0.695**

N = 290. *p < 0.05, **p < 0.01, (2-tailed). The bold values are average variance extracted.

### Hypothesis Test

This study used hierarchical regression analyses to test the research hypothesis through SPSS 24 software. SPSS allows researchers to test not only indirect effects but also mediated moderating effects, and has been adopted in entrepreneurship research (Liu et al., 2019). In order to verify whether EMC can positively affect IB, this research sets IB as the dependent variable, and results are presented in Table 4. In the first step, gender, age, education background, and time in the startup were setted up as control variables to exclude the impact of demographic variables on IB in the Model 1. Then, EMC was added as an independent variable, and Model 2 showed that EMC had a significant positive effect on IB (β = 0.347, *P* < 0.001), thus H1 was supported.

**TABLE 4:** Regression analysis of hypotheses.

Variables	Innovation behavior								Entrepreneurial self-efficacy	
	Model 1	Model 2	Model 3	Model 4	Model 5	Model 6	Model 7	Model 8	Model 9	Model 10
(1) Gender	–0.021	–0.024	–0.013	–0.014	–0.017	–0.001	0.008	–0.006	–0.072	–0.074
(2) Age	0.025	0.024	0.016	0.032	0.028	0.009	0.02	0.014	0.056	0.055
(3) Education background	–0.025	0.010	0.003	0.003	0.003	–0.031	–0.031	–0.040	0.021	0.050
(4) Experience in the startup	–0.057	–0.055	–0.048	–0.070	–0.052	–0.043	–0.063	–0.054	–0.049	–0.048
(5) EMC		0.347***	0.305***	0.275***	0.331***					0.281***
(6) ESE			0.149***			0.289***	0.250***	0.222***		
(7) ZYT				0.406***	0.377***		0.515***	0.495***		
(8) EMC × ZYT					0.265**					
(9) ESE × ZYT							0.234**			
*R* ^2^	0.006	0.306	0.336	0.434	0.470	0.134	0.357	0.376	0.012	0.139
Adjusted *R*^2^	–0.008	0.294	0.322	0.422	0.457	0.118	0.344	0.36	–0.002	0.124
*F*	0.435	25.063***	23.831***	36.194***	35.703***	8.753***	26.214***	24.265***	0.848	9.185***

N = 290. *p < 0.5, **p < 0.01, ***p < 0.001 (2-tailed).

Similarly, in order to verify the relationship between the EMC and ESE, this paper sets ESE as a new dependent variable. As shown in the Model 9, demographic variables were controlled to exclude possible substitution effects on ESE. On this basis, EMC was added into Model 10, and results showed that the correlation coefficient of EMC on ESE was 0.281, and significant at 0.001 level. H2 hypothesis was verified.

Furthermore, we tested the mediating effect of ESE by judging the following three conditions, namely, (1) EMC is significantly correlated with IB, (2) EMC is significantly related to ESE, and (3) when ESE is included in the relationship between EMC and IB, it is a complete mediation if the relationship between EMC and IB is not significant, but ESE and IB are significant. Otherwise, ESE plays a partial mediating role when EMC and IB are still significant but the correlation coefficient decreases. Condition 1 and 2 have been supported in Models 1 and 2, and Model 3 further revealed that ESE influence innovation behavior significantly (β = 0.149, *P* < 0.001), and the coefficient between EMC and IB (β = 0.305, *P* < 0.001) is decreased compared with Model 2 (β = 0.347 < 0.305). Thus, ESE partially mediates the relationship between EMC and IB, and H3 was supported.

**FIGURE 2:**
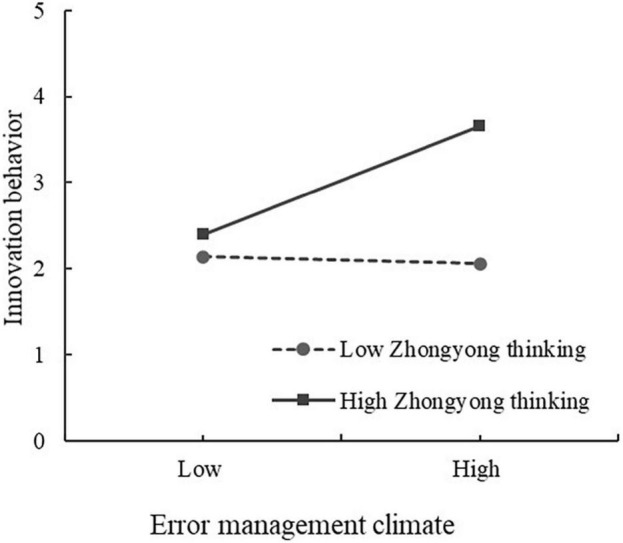
Moderating role of Zhongyong thinking in the relationship between error management climate and innovation behavior.

The last was to test the moderating effect of Zhongyong thinking. We mean-centered independent and moderator variables reduce potential multicollinearity problems (Aiken and West, 1991). Meanwhile, we constructed the interaction effect between EMC and ZYT (EMC). Before EMC × ZYT was considered, ZYT also had a positive correlation with IB (β = 0.406, *P* < 0.001). Then, Model 5 indicated that EMC × ZYT is significant with IB (β = 0.265, *P* < 0.01), suggesting that ZYT plays a moderating role in the relationship between EMC and IB. This study also performed simple slope analysis to verify further our research findings (Aiken and West, 1991). For individuals high on Zhongyong thinking (one standard deviation above the mean), a positive relationship between EMC and IB was found (*t* = 9.619, *p* < 0.001). As shown in Figure 2, for low ZYT individuals, ESE had a lower relationship with IB but not significant (*t* = −0.056, ns). Therefore, the relationship between EMC and JB is positively moderated by Zhongyong thinking. Thus H4 was supported.

**FIGURE 3:**
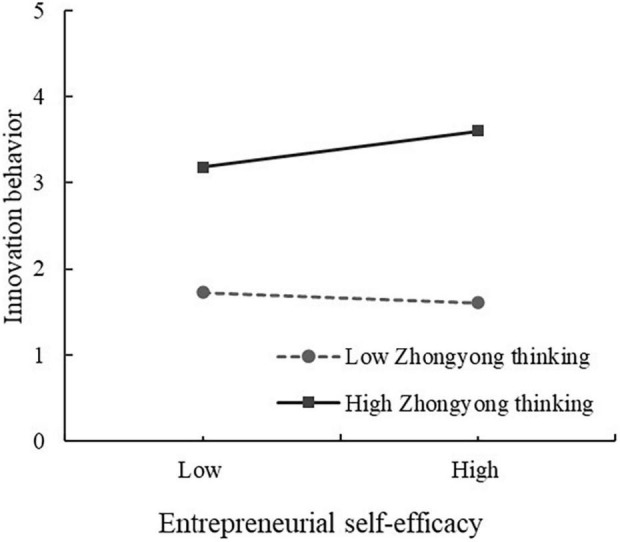
Moderating role of Zhongyong thinking in the relationship between entrepreneurial self-efficacy and innovation behavior.

Using the same research procedure, Model 8 demonstrated that the interaction between ESE and ZYT (ESE × ZYT) positively impacts IB (β = 0.234, *P* < 0.01). Simple slope analysis showed that ESE is more positively associated with IB (*t* = 5.513, *p* < 0.001) when entrepreneurs had higher levels of ZYT in Figure 3, thus, it supported H5a. Finally, we analyzed the conditional indirect effect of a relationship mediated by ESE, and bootstrapping analysis with 5,000 samples was conducted at 95% confidence interval. The analysis results (Table 5) revealed that indirect effect of EMC on IB through ESE was not significant (indirect correlation = −0.069) at the lower levels of ZYT, and the confidence interval was (−0.320, 0.183). When individual ZYT was at a high level, the indirect effect of EMC on IB through ESE was 0.138 (*p* < 0.05), and the confidence interval is (0.021, 0.254), CI did not include zero. In the case of high and low ZYT, the difference value of ESE between EMC and IB is −0.207 (*P* < 0.05), indicating that the impact of EMC influences IB *via* ESE is moderated by ZYT, and H5b was partially supported.

**TABLE 5:** Bootstrapping estimates for mediated moderating effect.

ZYT level	Moderator variable	Estimate	*SE*	Low 95% CI	High 95% CI	*P*
Low	EMC → ESE → IB	−0.069	0.123	−0.32	0.183	0.581
High		0.138	0.057	0.021	0.254	0.022
Difference		−0.207	0.074	0.013	0.206	0.037

*The coefficients in the table are non-standardized coefficients. 5,000 bootstrapping samples.*

## Conclusion

Modern enterprises increasingly rely on innovation to gain competitive advantages in a highly competitive market environment. How to stimulate and enhance innovation behavior is also an essential topic in the field of entrepreneurship. In recent years, error management has received attention in several areas because errors may hide improvable ways for better performance (Cope, 2011). However, there is still a lack of understanding about how errors become the source of innovation in entrepreneurship, and there is even less empirical evidence in the Chinese context.

This study first provides evidence that EMC has a positive effect on IB of entrepreneurs. Since errors are inevitable in entrepreneurship, what is more important for entrepreneurs is what they can learn from errors compared to the adverse effects after mistakes. The management climate for errors in the workplace influences entrepreneurs’ expectations of errors during innovation activities (Baer and Frese, 2003). Based on the positive perception of errors, EMC is conducive to the entrepreneur’s error thinking, error communication, error learning, and error ability (van Dyck, 2009). Therefore, EMC can be considered a kind of climate to encourage the atmosphere to convert errors into advantages. For individual innovation behavior in entrepreneurship, this study provides evidence that EMC is beneficial to reasonable errors handling and conducive to innovation behavior.

Secondly, this research also demonstrates that ESE as a mediator in the relationship between EMC and IB. Under a positive climate of error management, entrepreneurs can communicate with partners about causes of errors and learn from each other. EMC contributes to improve the identification, prevention, and timely correction of errors, and promote active behavior of error learning (Cigularov et al., 2010) so as to enhance the courage and confidence of self-exploration and innovation continuously. Thus, ESE can be obtained, modified, and enhanced as an important psychological resource in the process of translating errors into advantages. Furthermore, research evidence also reveals that entrepreneurs with higher ESE have a greater possibility of setting an innovative task goal and tackle the challenges coming from innovation (Chen and Zhou, 2017).

In addition, this paper proposed that ZYT plays moderating roles in the process of EMC influencing IB. As a typical characteristic of how Chinese view things, people, and environment, ZYT contributes to cope with entrepreneurial pressure, integrate resources, and implement appropriate methods, promoting the effective role of EMC and thus contributing to innovation behavior. Moreover, ZYT is conducive to the accumulation of ESE, and further beneficial for entrepreneurs to adopt appropriate ways to carry out entrepreneurial activities, enhancing the process of ESE influencing IB. Our empirical research also shows that ZYT moderates the direct and indirect relationship between EMC and IB.

## Discussion

### Theoretical Contribution

Firstly, this paper empirically studies the relationship between EMC and innovation behavior, providing empirical evidence from China since Chinese entrepreneurs play an important role in global innovation activities (Wei et al., 2020). Previous studies explained innovation behavior by demonstrating the impact of social capital and institutional factors (Stephens et al., 2013; Chen and Zhou, 2017), but there is a lack of study carried out from the perspective of EMC. Based on verifying the positive side of entrepreneurial errors (Fischer et al., 2018), this study explored how EMC influences IB. Our findings are consistent with the view that errors can be opportunities for organizational learning and innovation apart from the negative results, indicating that error tolerance of the enterprise and good EMC is conducive to the innovation behavior of entrepreneurs. At the same time, this paper improves the objective understanding of errors and the importance of effective error management, providing a useful reference for further detailed research on error management in the entrepreneurial field.

Secondly, this paper demonstrates the mediating effect of ESE, a specific self-efficacy concept applied in entrepreneurship (Chen and Zhou, 2017), to answer the question about how error becomes the source of innovation. Errors can be correctly recognized and learned, thus positive-oriented EMC plays an important role in promoting ESE and further influences IB, increasing the general cognition that increased psychological resources can promote innovation behavior. This corresponds to the growing evidence that psychological capital influences a series entrepreneurial outcomes, such as innovation behavior and enterprise performance (Hechavarria et al., 2012; Gao et al., 2020). Compared with institutional and economic factors, personal characteristics, especially psychological capital factors, has attracted more attention since researcher advocate that individual characteristics of entrepreneurs determine the degree of success in the entrepreneurship (Altinay and Wang, 2011). This paper answers the research call of Chen et al. (1998) about consideration on the application of ESE in more contexts by enriching the mechanism research in the Chinese context.

Thirdly, this paper adds to the understanding of the Chinese indigenous research perspective by examining moderating roles of ZYT. Theoretical discussions on traditional “west type” entrepreneurship theory basically assume that entrepreneurship prevails only in cultures dominated by individualism and in societies that encourage individual risk-taking and self-actualization (Erez and Gati, 2004; York and Venkataraman, 2010). However, researches have witnessed that a large number of countries with collectivist cultures, such as China and India in Asia and Zimbabwe in Africa and Chile in South America, having more active entrepreneurial activities than many Western countries with individualistic cultures in recent 20 years (Ibrayeva, 2006; Hampel-Milagrosa et al., 2015), which makes people doubt the applicability of the previous theories. We speculate that this stereotype may have arisen because previous research samples were mainly limited to developed countries. Relating to entrepreneurs’ personal traits and cultural background, traditional ZYT feature actually helps entrepreneurs to manage risks and uncertainties in the Chinese context, which is reflected in prevention before errors and positive adjustment after errors happen, preventing innovation activities from sliding into the extreme. Similar to the conclusion that innovation is related to the cultural practice of error management from Fischer et al. (2018), this paper provides empirical experience from China on how ZYT plays a positive role in error management and innovation activities, proving that ZYT can be considered as a cognitive strategy to cope with errors and entrepreneurial risks nowadays effectively.

### Practical Implications

In practice, our research findings also provide some potential implications. This paper firstly suggests that entrepreneurs should objectively and correctly face up to the errors in the process of entrepreneurship and promote a good climate of error management referring to error communication, reflection, and learning. For novice entrepreneurs, they need to be aware of the risks of innovation activities before starting a business, so that they can be psychologically prepared to deal with a series of errors that may deviate from their goals. Meanwhile, a recovery path for entrepreneurs who are experiencing entrepreneurial errors has been proposed. Errors need to be dealt with in a certain way to allow innovations to develop (Fischer et al., 2018). The key of turning error experience into advantages depends on their ESE and ability to learn from errors. Awareness of this path may help entrepreneurs pay more attention to the error itself rather than negative emotional responses.

Secondly, entrepreneurs are suggested to enhance their confidence in completing activities related to entrepreneurship by continuous learning, practicing, and reflecting, thus improve their psychological capital and conduct innovative behavior. Innovation is a kind of entrepreneurial activity requiring extensive and effective use of various resources. Apart from tangible resources, entrepreneurs also need to accumulate more intangible resources, such as entrepreneurial knowledge, self-efficacy, and self-regulation ability (Attour and Lazaric, 2020).

Thirdly, entrepreneurs can regard ZYT as a cognitive strategy to cope with errors effectively. ZYT is not only one of the representative modes of thinking in Chinese people, but also has a broad practical basis in entrepreneurship. Our research is helpful for Chinese entrepreneurs to objectively understand the positive aspects of traditional culture and encourages cross-cultural entrepreneurs to consider problems from multiple perspectives, integrate various elements, and carry out innovative activities in an appropriate way, making cross-cultural communication and cooperation possible. In addition, schools and government agencies also play important roles in supporting innovation. Education about entrepreneurial errors and activities to cultivate ZY can be carried out in schools for potential entrepreneurs. Besides policy support, government agencies can coordinate with schools to establish error communication centers or mutual aid associations, where entrepreneurs can restore and accumulate psychological capital by exchanging error experiences.

### Limitations and Future Research

Although this paper contributes to the understanding of how EMC affects innovation behavior in the Chinese extent, some limitations still need to be noticed. The first is to optimize measurement methods. The questionnaire survey was conducted in the form of a self-report by entrepreneurs. Although the results of the standard method bias test showed that social approval and other issues would not seriously affect the validity of conclusions, future research can adopt the measurement method of multi-subject paired questionnaire or conduct situational experiments. The second is to strengthen the universality of research samples and carry out longitudinal tracking studies. Nearly half of the participants in this study were from Jiangsu Province, which may have regional biases and the data are cross-sectional. Since entrepreneurs need some time to recover and learn from errors once they happen, future research can be carried out on a broader range of samples and longitudinal follow-up to collect data from different regions and periods. This paper explores the mediating role of ESE and the boundary influence of ZYT based on the theory of resource conservation. In the future, more influencing mechanisms can be considered from the perspectives of individual traits, knowledge exchange, and emotional state. At the same time, detailed studies on error management can be carried out to explore the possible differential influence paths between the EMC and the error aversion climate. In addition, the ZYT scale in this study was generated in the Chinese cultural context, and the universality of the scale needs further verification considering cultural factors. Although ZYT is a typical Oriental cultural characteristic, cross-cultural entrepreneurs can learn and exercise it as a positive cognitive strategy. Future research opportunities come from the comparative study of ZYT in different cultures and the model’ s applicability in other cultural contexts.

## Data Availability Statement

The raw data supporting the conclusions of this article will be made available by the authors, without undue reservation.

## Author Contributions

YC participated in the design and drafting of the early version and data analysis. JW participated in the design and drafting of the early version and revising of the manuscript. JZ and XL participated in the data analysis and revise of the manuscript. All authors contributed to the article and approved the submitted version.

## Conflict of Interest

The authors declare that the research was conducted in the absence of any commercial or financial relationships that could be construed as a potential conflict of interest.

## Publisher’s Note

All claims expressed in this article are solely those of the authors and do not necessarily represent those of their affiliated organizations, or those of the publisher, the editors and the reviewers. Any product that may be evaluated in this article, or claim that may be made by its manufacturer, is not guaranteed or endorsed by the publisher.

## Appendix

**TABLE TA1:** Measurement instruments for variables.

Reflective construct	Standardized loadings (λ)*
**Error management climate (AVE = 0.757; CR = 0.956)**	
For us, errors are very useful for improving the work process	0.756
After making a mistake, people try to analyze what caused it	0.818
Our errors point us at what we can improve	0.847
When mastering a task, people can learn a lot from their mistakes	0.824
When people are unable to correct an error by themselves, they turn to their colleagues	0.831
When someone makes an error, (s)he shares it with others so that they do not make the same mistake	0.847
In this organization, people think a lot about how an error could have been avoided	0.848
**Entrepreneurial self-efficacy (AVE = 0.735; CR = 0.860)**	
I know about startups and the activities during entrepreneurship	0.756
I can balance different points of view and resolve team conflicts	0.770
I can come up with new ideas to solve problems in entrepreneurship	0.737
I have confidence in my ability to solve problems in my business	0.758
**Innovation behavior (AVE = 0.674.; CR = 0.939)**	
I always seek to apply new processes, techniques and methods	0.755
In order to implement new ideas, I can find ways to get the resources I need	0.663
In order to realize new ideas, I can make suitable plans	0.668
I often come up with creative ideas	0.732
I often communicate with others and present my new ideas	0.672
Generally speaking, I am an innovative person	0.687
**Zhongyong thinking (AVE = 0.695; CR = 0.967)**	
When discussing, I will consider the conflicting opinions at the same time	0.734
I often think about the same thing from different perspectives	0.607
I will listen to all the opinions before I express them	0.649
When I make a decision, I will consider various possible conditions	0.666
I often try to find acceptable opinions in a situation of disagreement	0.709
I often try to find a balance between my own opinions and those of others	0.683
I will adjust my original ideas after considering the opinions of others	0.678
I expect to reach a consensus during the discussion	0.669
I try to incorporate my own opinions into the thoughts of others	0.715
I usually express conflicting opinions in a tactful way	0.657
I will try to reconcile the minority to accept the majority in a harmonious way	0.642
I usually consider the harmony of the organizational climate before making a decision	0.698
I usually adjust my behavior for overall harmony	0.663

**All standardized loadings are significant (p < 0.01).*
